# Strategy for improving extracellular lipolytic activities by a novel thermotolerant *Staphylococcus *sp. strain

**DOI:** 10.1186/1476-511X-10-209

**Published:** 2011-11-11

**Authors:** Slim Cherif, Sami Mnif, Fatma Hadrich, Slim Abdelkafi, Sami Sayadi

**Affiliations:** 1 Laboratoire des Bioprocédés Environnementaux, Pôle d’Excellence Régional (PER, AUF), Centre de Biotechnologie de Sfax, Université de Sfax, Tunisia

## Abstract

**Background:**

Extracellular bacterial lipases received much attention for their substrate specificity and their ability to function under extreme environments (pH, temperature...). Many staphylococci produced lipases which were released into the culture medium. Reports of extracellular thermostable lipases from *Staphylococcus *sp. and active in alkaline conditions are not previously described.

**Results:**

This study focused on novel strategies to increase extracellular lipolytic enzyme production by a novel *Staphylococcus *sp. strain ESW. The microorganism needed neutral or alkaline pH values between 7.0 and 12.0 for growth. For pH values outside this range, cell growth seemed to be significantly inhibited. *Staphylococcus *sp. culture was able to grow within a wide temperature range (from 30 to 55°C). The presence of oils in the culture medium leaded to improvements in cells growth and lipolytic enzyme activity. On the other hand, although chemical surfactants leaded to an almost complete inhibition of growth and lipolytic enzyme production, their addition along the culture could affect the location of the enzyme. In addition, our results showed that this novel *Staphylococcus *sp. strain produced biosurfactants simultaneously with lipolytic activity, when soapstock (The main co-product of the vegetable oil refining industry), was used as the sole carbon source.

**Conclusion:**

A simultaneous biosurfactant and extracellular lipolytic enzymes produced bacterial strain with potential application in soap stock treatment

## Background

Lipolytic enzymes catalyse hydrolysis and synthesis reactions, either in long chain triacylglycerols (lipases) or in short chain fatty acids (esterases) [1], most of them of industrial interest in areas such as food, detergent, paper or oleochemical industries. Nowadays, there has been an increasing interest in the study of enzymes from extremophiles, since they are not only more thermostable but often more resistant to chemical agents and extreme pH values than their mesophilic homologues [2-4]. The production of microbial lipases has been shown to be influenced by several factors, namely the carbon source, temperature, pH, dissolved oxygen concentration and presence of inducers. These compounds, such as oils and some surfactants, have been described as agents that increase the production of enzymes with lipolytic activity. Also, in some cases they are essential for lipolytic activity to be detected [4]. Last, the engineering of culture conditions has also been shown to be an effective mode to achieve enzyme preparations enriched in selected isoenzymes which are effective for particular biotechnological applications [5]. In previous paper [6-8], many authors show that enzyme production was not fully associated to growth rate, although absolute values of total lipolytic activity and biomass were positively correlated. However, cell growth was relatively low, and lipolytic activity appeared to be largely retained within the biomass. Therefore, it would be interesting to find culture conditions (i.e. medium composition, pH, temperature, aeration), allowing to improve growth and/or favour enzyme secretion. In this work, optimisation of lipolytic enzyme production by a newly isolated *Staphylococcus *sp. strain has been attempted. The influence of incubation temperature, the effect of pH on the growth and enzyme production, and the influence of some other parameters in the culture medium have been investigated. Finally, *Staphylococcus *sp. culture was found to be able to grow on soapstocks (one of the major by-products from vegetable oil refining). This rich bacterial substrate was found to be soluble, when biosurfactants and lipolytic enzymes were produced in the culture medium.

## Methods

### Microorganism

The identification of the bacterial strain ESW has been previously determined in our laboratory. The methods used for 16S rRNA gene amplification and sequencing have been previously reported [9,10]. Sequence data were imported into the sequence editor BioEdit version 5.0.9. The full sequence was aligned using the RDP Sequence Aligner program [11]. The consensus sequence was manually adjusted to conform to the 16S rRNA secondary structure model. Sequences used in the phylogenetic analysis were obtained from the RDP and GenBank databases [11,12]. Positions of sequence and alignment ambiguity were omitted andpairwise evolutionary distances were calculated using the method of Jukes and Cantor [13]. Strain ESW was affiliated to *Staphylococcus *genus and designed as *Staphylococcus *sp. strain ESW.

### Culture medium

The microorganism was grown in a liquid medium containing per liter: 5 g yeast extract, 10 g NaCl, 10 g peptone. The medium was autoclaved at 121°C for 20 min. Cultures were carried out in 250 mL Erlenmeyer flasks with 50 mL of medium. Moreover, some experiments were realized in culture media composed only by soapstocks (by-products of vegetable oil industry).

### Inducer effect

Several experiments were carried out to determine the inducing effect by adding 1% of several oils and surfactants, namely olive oil, soybean oil, trioctanoin, tributyrin, Triton X-100 or Tween 80, to the flasks at the beginning of the cultures. Moreover, the best inducer was tested by adding this compound at 0, 5 or 10 h of growth in order to study the effect of addition time. Samples were taken during the stationary phase after 30 h of growth. Furthermore, the effect of surfactants (Triton X-100, Tween 80 and 20, CHAPS and PEG 200) was also tested by adding each of them at the beginning of the stationary phase after 24 h of growth. Samples were taken immediately before and after the addition of the surfactant and at the 30 h of growth.

### Analytical methods

#### Sample preparation

Cells were harvested by centrifugation (10 min, 5000 g) and suspended in 4 ml Tris/HCl buffer 50 mM pH 7.5, containing 25 mM EDTA and 25 mM NaCl. The supernatant was reserved for extracellular enzyme analysis. The cell suspension was sonicated in two cycles of 2 min at 50% of the maximum power (Branson Sonifier, model 250). The procedure was carried out in an ice bath, and a 2 min cooling time was allowed between cycles. Then, the mixture was centrifuged for 10 min at 5°C and 5000 g. The supernatant and the pellet were kept for the measurement of intracellular lipolytic activity and of membrane lipolytic activity, respectively.

#### Cell growth determination

Biomass concentration was measured *via *turbidimetry at 600 nm and the obtained values were converted to concentration by using a previously determined calibration curve.

#### Lipolytic activity assay

The lipase activity was assayed by measuring the free fatty acids released from mechanically stirred emulsions of triacylglycerols, using 0.1 N NaOH with a pH-Stat (Metrohm, Switzerland). The kinetic assay was performed, in optimal conditions (pH 12.0 and 60°C) using 0.25 ml TC_4_ (Sigma) in (30 ml 2.5 mM Tris-HCl, 150 mM NaCl and 0.5 mM Sodium deoxycholate (NaDC)) or in olive oil emulsion obtained by mixing (3×30 s in a Waring blender), 10 ml of olive oil (Sfax-huile, Tunisia) in 90 ml of 10% GA (Gum Arabic). One lipase unit corresponds to 1 μmol of fatty acid released per minute [14].

#### Biosurfactant production determination

Surface tension measurement was used to evaluate biosurfactant production when soapstosks was used as culture medium. Samples of the culture media of the selected strains were centrifuged at 8000 xg for 20 min. Surface tension (ST) of the supernatant fluid of the culture was measured by the ring method using a DuNouy ring tensiometer (Kruss T 10, Hamburg, Germany).

## Results and discussion

In order to improve growth and/or favour the enzyme secretion, the influence of some key variables such as medium composition (i.e., ion concentrations of mineral water, lipid compounds, surfactants) and culture conditions (i.e., pH, temperature, visible radiation), were assessed. From the preliminary results obtained, it can be concluded that when *Staphylococcus *sp. was grown in the presence of visible light, no significant changes in the parameters studied were detected. Moreover, although a previous paper [15] confirmed that ion concentrations in mineral water (Na^+^, Ca^2+^, Mg^2+^ and HCO_3_^-^) comprise the ions which are, most probably, responsible for stimulating the lipolytic activity, the use of mineral concentrated water did not improve either cell growth or lipolytic enzyme secretion. On the other hand, some factors such as culture pH, culture temperature and the addition of lipid compounds and surfactants seemed to have great influence on the behaviour of the microorganism. Hence, these variables are studied in more detail in this work. An operational classification of enzyme activities as intracellular, extracellular or membrane lipolytic activity has been utilised. Thus, activity detected in the culture medium after biomass separation by centrifugation was considered as extracellular, while that recovered in solution after sonication of the buffer-resuspended cells and elimination of cell debris was considered as intracellular and membrane lipolytic activities, respectively [16,17].

### Effect of pH

**Figure 1:**
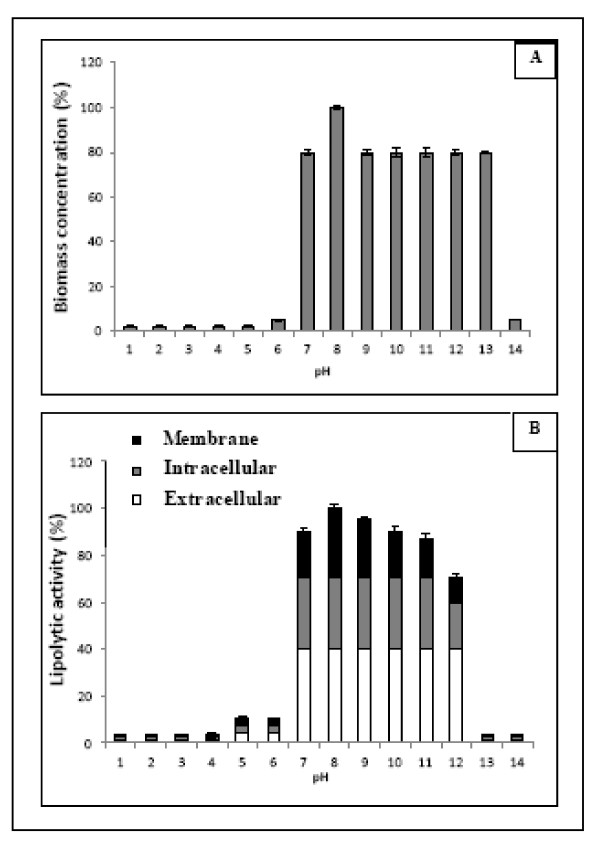
**Effect of initial pH culture medium on biomass concentration**. (A) and lipolytic activity (B) of *Staphylococcus *sp. The values are shown in percentage of the maximum values.

The effect of pH on the growth and enzyme production by *Staphylococcus *sp. was assessed in a wide range (4.0-13.0). The maximum values of biomass concentrations and lipolytic activities obtained after 30 h of growth are showed in Figure 1. From these results, it was concluded that the microorganism needs neutral or alkaline pH values between 7.0 and 12.0. For pH values outside this range, cell growth seems to be completely inhibited a fact which reveals the importance of studying this factor in cultures of *Staphylococcus *strain and of controlling the pH variations during its cultivation. As it was demonstrated in a previous paper [15], the enzymatic synthesis can be greatly associated with cell growth. Thus, the highest values of enzyme production, maximum at pH 8.0, were also reached in the same range of pH values where the microorganism showed optimal growth. Moreover, it was detected that an important percentage of the produced enzyme was located on the cell membrane, while the extracellular enzyme represented only about 40% of the total enzyme. This fact, that has also been found in previous work [16], indicates that an important step to be undertaken in this time of work is to find adequate operating strategies, such as the addition of substances to the culture medium, that can favour the release of this bound enzyme to the medium and facilitate the recovery method; thus, the expensive processes that involve cell lysis are avoided.

### Effect of temperature on growth and lipolytic activity

The studied strain was grown in shake flasks, within a wide range of temperature (from 30 to 55°C). The increase in temperature seemed to have a negative effect on biomass production and also lipolytic activity. Biomass production and total lipolytic activity reached their maximum, in flask culture incubated at 30°C after 24 h of incubation. For flask cultures incubated at temperature higher than 30°C, biomass production and total lipolytic activity were negligible after the same incubation time. The maximum biomass production and the total lipolytic activity levels were measured for 40 and 55°C at 48 h and 72 h of incubation, respectively (data not shown). One can say that in all cases, the highest final values were obtained when operating at a wide temperature range from 30 to 55°C. Cultures showed a significant decrease in cell growth and lipolytic activity at temperatures above 55°C. The cultures were stopped after 24 h of incubation at 30°C, since previous experiments indicated that, no significant increases in lipolytic enzyme activity were attained later in flask cultures [18].

### Influence of lipid compounds and surfactants

**Figure 2:**
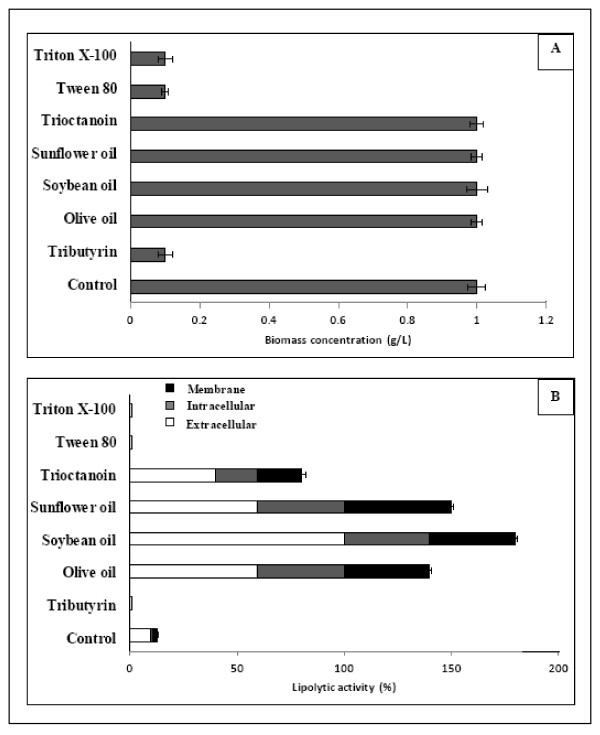
**Influence of the presence of different lipid and surfactant compounds on biomass concentration**. (A) and lipolytic activity production (B) of *Staphylococcus *sp. The data concerning lipolytic activity are shown in percentage of the control culture after 24 h of growth.

The addition of lipid compounds to the culture medium generally favours the production of lipolytic enzymes. The most widely used lipid inducers in these processes are fatty acids, triacylglycerols and some esters [19-21]. Therefore, the next step in this work was to study, on the one hand, the influence of the addition of different compounds on the lipolytic production of *Staphylococcus *sp., and on the other hand, the moment at which these compounds should be added. On the first approach, culture media with a concentration of 1% of different lipids and surfactants were used. The selection of this value was based on the improvements of lipolytic enzyme production attained in several previous papers [22,23]. The lipids chosen in this work were olive oil, soybean oil, sunflower oil, trioctanoin and tributyrin. The surfactants chosen were Tween 80 and Triton X-100. As indicated in Figure 2, the effect over biomass concentration depends on the tested compound. While the addition of long and medium chain triacylglycerols (represented by olive oil, soybean oil, sunflower oil, and trioctanoin) seems to produce no observable variation in comparison with the control culture, growth was completely inhibited in the presence of short chain triacylglycerols (tributyrin) and surfactants (Tween 80 and Triton X-100); in this way, lipolytic enzyme production was prevented. These surfactants have been referred to, in the literature, as inducers of lipolytic enzyme production. Tween 80 has proven an inducing effect on *Aspergillus terrus*, *Candida cylindracea *and *Serratia marescens *[24-26], while Triton X-100 has shown beneficial effects in the case of *Hendersonula toruloidea *[25]. However, there are also examples of negative effects, some of which are shown here. In fact, Lima et al. [27] found that the addition of Tween 80 led to a total inhibition of cell growth, while Triton X-100 originated a decrease of 50%. Likewise, Lin et al. [28] observed no enzymatic activity after addition of Tween 80 and Triton X-45 in cultures of *Pseudomonas pseudoalcaligenes *F-111. On the other hand, the addition of the selected oils generated different effects over enzymatic production (Figure 2). Trioctanoin did not lead any improvement in total lipolytic enzyme production; in fact, it even seemed to involve a decrease in production. The best results were obtained when sunflower oil, olive oil and soybean oil were added. These led to an increase of about 80% in enzymatic production when compared with the value obtained in the control culture. In studies about several lipase producing microorganisms, such as *Pseudomonas, Geotrichum candidum, Candida rugosa, Aspergillus fumigatus, terrus and niger, and Bacillus coagulans *[21,25,29-32], the oils above mentioned have also been considered to be good inducers of lipolytic activity. Oleic acid has been recently considered as one of the most important substrates for lipase production by *Rhizopus chinensis *[33]. A study dealing with lipase production by *C. rugosa *has shown that olive oil and oleic acid induce the translation of the genes that code for lipolytic enzymes [5]. Another interesting observation concerns the location of the lipolytic enzymes produced by *Staphylococcus *sp.. It was detected that growth in the presence of sunflower oil leads to a higher proportion of extracellular enzyme. Hence, from here onwards this paper focuses on a more detailed study of this inducer. In particular, the effect of the time point of addition of sunflower oil on lipolytic enzyme production is considered.

### Influence of the time of inducer addition

**Figure 3:**
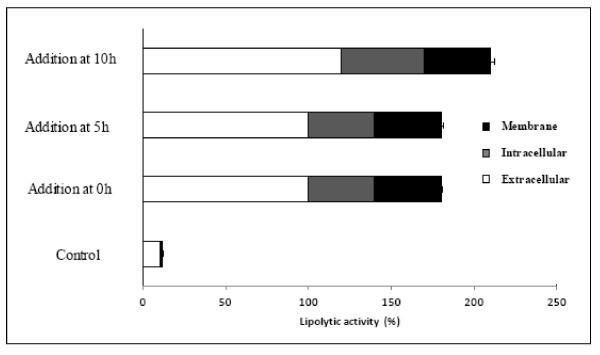
**Effect of the time of addition of sunflower oil on the lipolytic activity of *Staphylococcus *sp.**. The data of lipolytic activity were taken after 24 h of growth and shown in percentage of the control culture.

In the above experiment, the addition of an inducer was performed at the beginning of the cultivation. It is common practise to add the principal components of the culture broth, such as inducers or carbon sources, at different times during the biological process. However, in the literature, when the addition of lipolytic inducers into the culture medium is undertaken, these inducers are added at different culture times depending on the authors. Nonetheless, it is not clear whether such times of addition are optimal. Zhang et al. [34] studied the most effective time for inducer addition to *C. rugosa *cultures. They observed that addition of Tween 80 at an earlier period of cultivation (0 or 6 h) was more effective than at a later stage (18 h). Another study [35] with *C. rugosa *compared a culture where oleic acid (as a carbon source) was added constantly throughout the culture with another culture with specific growth rate control. This study showed that different strategies of feeding oleic acid to the culture lead to different profiles of isoenzymes, and that by working with specific growth rate control, higher extracellular activities can be obtained. Thus, the next step of our work was to study the effect of adding sunflower oil, considered as the best inducer, to the culture broth at different times. Several cultures were prepared with addition of sunflower oil at the beginning of the culture, at the end of the lag phase (5 h) or after the beginning of the exponential phase (10 h). The results are shown in Figure 3. Although, sunflower oil addition at different times did not lead to large differences regarding lipolytic activity, the levels of enzymatic production when sunflower oil was added after 10 h of growth were slightly higher. Therefore, addition will be carried out at the beginning of the exponential phase in future experiments.

### Using surfactants to favour the release of membrane bound Enzyme

Biological membranes consist of a bilayer of lipids with two kinds of proteins: intrinsic or integral proteins, which are embedded in the bilayer, and peripheral proteins, which are adsorbed to the membrane. Surfactants have the ability to solubilise the lipids on the membrane forming micelles and extracting membrane bound proteins [36]. For this reason, although it has been previously reported that surfactants inhibit *Staphylococcus *sp. growth, it could be interesting to verify the influence of surfactant addition at the stationary phase of the culture and to evaluate the ability of surfactants to promote an increase in the extracellular lipolytic activity proportion. Accordingly, the effect of the addition of five different surfactants (polyethylene glycol with a molecular weight of 200 (PEG 200), Tween 80, Tween 20, CHAPS and Triton X-100) on the level of extracellular lipolytic activity was evaluated. Our results show that a large increase in extracellular lipolytic activity in all cases, except with PEG 200. PEG 200 did not lead to significant changes when compared to control, possibly due to the lower detergent ability of this compound (data not shown). As it has been previously mentioned, surfactants have the ability to solubilise lipids and proteins of the cell membrane. Thus, it is possible that disruption of the cell membrane, in the presence of these surfactants, might have provoked a partial cell lysis. Our results reveal a release of intracellular lipolytic enzymes that seems to confirm this fact. In order to confirm this hypothesis, it is worth to analyse the levels of biomass concentration after introducing these compounds into the culture medium. Table 1 shows the biomass concentration before and after surfactant addition as well as 6 h after addition. Except for PEG 200, which showed no significant effect, all the surfactants caused a decrease in biomass concentration. This effect was particularly noteworthy in the cases of CHAPS and Triton X-100, as it could have been expected from the data of lipolytic activity distribution. Then, the lytic effect of these substances was confirmed. However, Tween 80 apparently caused less extreme cell damage since both intracellular activity and biomass concentration suffer a less prominent decrease.

**Table 1:** Effect on biomass of *Staphylococcus *sp. by the addition of several surfactants after 24 h of growth.

Surfactants	Biomass-before addition (g L^-1^)	Biomass-after addition (g L^-1^)	Biomass-30 h (g L^-1^)
Control	1.01 ± 0.04	1.00 ± 0.02	1.06 ± 0.01

CHAPS	1.01 ± 0.03	0.65 ± 0.03	0.35 ± 0.03

Tween 80	1.00 ± 0.04	1.00 ± 0.04	0.80 ± 0.04

Tween 20	1.00 ± 0.02	0.95 ± 0.06	0.70 ± 0.02

PEG 200	1.01 ± 0.01	0.98 ± 0.03	1.06 ± 0.02

Triton X-100	1.00 ± 0.05	0.80 ± 0.04	0.25 ± 0.01

These results demonstrate that the compounds leading to a higher increase of extracellular lipolytic activity were Tween 80 and Triton X-100, each one by a different mechanism: the first by allowing a release of the membrane bound enzyme without causing too much cell damage, and the second by favouring lysis, which triggers the release of both membrane and intracellular protein. As a consequence, the extracellular lipolytic activity is considerably increased and, thus, it is not necessary to use another technique to achieve cell lysis (such as ultrasounds). Therefore, either one or the other surfactant could be selected depending on the operational system used as well as on the economic factors involved.

### Simultaneous production of lipolytic activity and biosurfactant when using soapstocks as the sole carbon source

**Figure 4:**
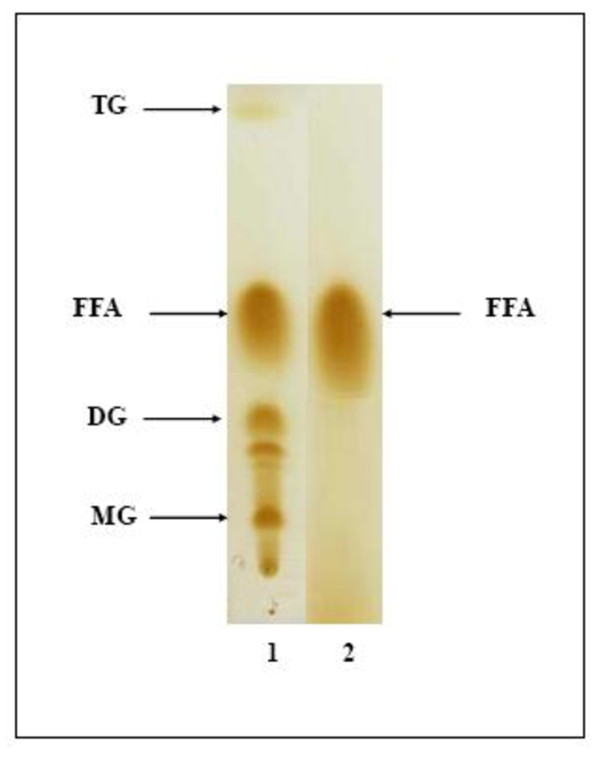
**TLC profile of the free fatty acids liberated after hydrolysis of soapstocks by *Staphylococcus *sp.. lipolytic enzyme**. Line 1: Soapstocks before lipolytic enzyme addition, Line 2: Soapstocks after lipolytic enzyme addition.

Microorganisms degrading various types of hydrocarbons are ubiquitous in nature [37]. Many of them usually produce potent emulsifiers and these surfactants help them to degrade insoluble substrates [38]. When *Staphylococcus *sp. strain was cultivated on soapstosks, the surface tension of the culture dropped rapidly till the 24 h of incubation to reach after several hours its lowest point which was about 25.7 mN/m. The diameter of the clear zone obtained by oil displacement test method was more than 8 cm (data not shown). The reduction of the surface tension of the culture indicated that biosurfactants were produced. Simultaneously, *Staphylococcus *sp. strain produces enzymes which enhance the degradation of soapstosks components, essentially a lipolytic activity. In fact, when this co-product (soapstocks) was only used as culture medium, lipolytic activity was detected and reached the maximum (data not shown). The co-produced biosurfactant plays an essential role for the solubulisation of soapstocks, which can be easily hydrolysed by produced lipolytic enzyme. In fact, soapstock constituted essentially by a mixture of TG (triglyceride), FFA (free fatty acid), DG (diglyceride) and MG (monoglyceride), whereas after hydrolysis by lipolytic enzyme and simultaneous solubilisation by co-produced biosurfactant, only FFA were present (Figure 4).

## Conclusion

The results obtained in this study permit to conclude that pH is a highly significant factor in growth of a newly isolated *Staphylococcus *sp. strain, with an optimal growth and lipolytic enzyme production at pH 8.0 and a wide temperature range (from 30 to 55°C). Moreover, the effect of the addition of several inducers on enzyme production shows a different behaviour. This novel bacterium *Staphylococcus *sp., isolated from soil, was found to produce biosurfactants when grown on soapstocks, a co-product of the vegetable oil refining industry, as the sole carbon source.

## Competing interests

The authors declare that they have no competing interests.

## Authors' contributions

SC, SM and FH designed the experiments, analyzed the data and drafted the manuscript. SA and SS conceived research and approaches and have given final approval of the manuscript to be published. All authors read and approve the final manuscript.
